# The Role of Psychopathy in Subtypes of Aggression and Gun Violence

**DOI:** 10.1007/s10862-025-10247-3

**Published:** 2025-09-02

**Authors:** Nicholas D. Thomson, Sophie L. Kjaervik, Craig S. Neumann, Robert D. Hare

**Affiliations:** 1 Department of Surgery, Virginia Commonwealth University, Richmond, VA 23284 USA; 2 Department of Psychology and Psychiatry, Virginia Commonwealth University, Richmond, VA 23284 USA; 3 Department of Psychology, University of North Texas, Denton, TX 76203 USA; 4 Department of Psychology, The University of British Columbia, Vancouver, BC V6T 1Z2 Canada

## Abstract

**Objective:**

This study examined the relationship between psychopathy and subtypes of aggression and firearm violence among a high-risk, community-based sample of adults. Specifically, it assessed whether the four-facet model of psychopathy (interpersonal, affective, lifestyle, and antisocial) was differentially associated with reactive and proactive aggression and reactive and proactive gun violence. Additionally, a confirmatory factor analysis (CFA) was conducted to evaluate the factor structure of the Self-Report Psychopathy Short Form (SRP-SF) in this population.

**Method:**

A total of 458 violently injured adults (M_age_ = 32.8, SD = 12.8, 72% Male) were included in this study. A CFA assessed the SRP-SF facet structure. Regressions were conducted to determine if psychopathy total and facets scores were associated with reactive and proactive aggression and gun violence.

**Results:**

Total psychopathy was associated with reactive and proactive forms of aggression and gun violence. The four-facet model had a good fit. Regressions showed that the affective and lifestyle facets were related to reactive aggression, and the interpersonal and antisocial facets were related to proactive aggression. Higher affective facet scores were associated with increased odds of reactive gun violence, while higher antisocial facet scores were associated with increased odds of proactive gun violence.

**Conclusion:**

The findings support the four-facet structure of psychopathy among a high-risk community sample and demonstrate its utility for differentiating violence subtypes. These results highlight the importance of considering psychopathy’s multidimensional nature in understanding specific risks for firearm-related violence, providing valuable insights for targeted violence prevention and intervention strategies within healthcare and community settings.

## Introduction

Psychopathy has long been recognized as one of the strongest predictors of violent behavior, making it a critical construct in forensic psychology, criminology, and public health (Hare, 2003; Olver et al., 2020; Thomson, 2019). People high on psychopathic traits exhibit a range of personality and behavioral characteristics that facilitate engagement in violent acts, including callousness, emotional detachment, manipulativeness, and a willingness to violate others without remorse (Neumann & Hare, 2008). The consequences of psychopathy-related violence extend beyond individual criminal acts to broader social implications, including increased victimization, higher rates of violent recidivism, and significant economic costs to the criminal justice and healthcare systems (Kiehl & Hoffman, 2011). Given these stakes, understanding the association between psychopathy and violence is essential for risk assessment and violence prevention strategies, particularly among high-risk populations. One such group includes violently injured individuals. These are community adults who have sustained injuries from interpersonal violence and are often overlooked in psychopathy research. Compared to the general population, violently injured individuals have extensive histories of violence perpetration and elevated risk for future violence (Kjærvik & Thomson, 2024). In one longitudinal study, 36% reported perpetrating a violent offense, including assault or firearm use, within two years of the initial injury (Cunningham et al., 2009). These individuals often present with multiple violence-related risk factors, including gang involvement, weapon carrying, and prior justice system contact, all of which contribute to their continued involvement in cycles of violence. Their risk of death from subsequent violence is approximately 20 times higher than that of non-injured individuals (Cunningham et al., 2009). Unlike incarcerated samples, they remain embedded in community settings, making them particularly relevant for research aimed at understanding how psychopathic traits contribute to real-world violence. Despite their elevated risk profile, psychopathy and its facets have rarely been examined in this high-risk population (Thomson et al., 2025).

Psychopathy was originally conceptualized as a unitary construct characterized by emotional detachment and superficial charm (Cleckley, 1941), but later bifurcated into personality and behavioral dimensions (Hare et al., 1990). More recently, factor-analytic research supports a four-facet model comprising interpersonal, affective, lifestyle, and antisocial traits (Hare & Neumann, 2008). The interpersonal facet reflects manipulativeness and grandiosity; the affective facet includes shallow affect and lack of remorse; the lifestyle facet captures impulsivity and irresponsibility; and the antisocial facet involves rule-breaking and aggression (Neumann et al., 2012). While interpersonal and affective traits represent more covert dissociality, the antisocial facet reflects overt antisociality (Hare & Neumann, 2009). Together, these dimensions reflect chronic dissociality (Neumann et al., 2007). This multifaceted structure has proven valuable across forensic and developmental contexts for understanding distinct correlates of antisocial behavior, aggression, and recidivism. (Blair, 2010; Coid & Yang, 2011; Olver et al., 2020; Salekin et al., 2004, 2018; Thomson, 2019).

Aggression is commonly distinguished into two subtypes, reactive and proactive subtypes (Dodge & Coie, 1987). Reactive aggression is impulsive and emotionally charged, often triggered by perceived provocation or threat and linked to poor emotion regulation and hostile attribution biases (Berkowitz, 1993; Raine et al., 2006). Proactive aggression, by contrast, is deliberate and goal-oriented, serving instrumental purposes such as dominance or gain, and is typically reinforced through learned reward contingencies (Berkowitz, 1993; Raine et al., 2006). These subtypes differ in their motivational and neurocognitive profiles and have distinct implications for understanding psychopathy-related violence (Thomson et al., 2021). Meta-analytic and community-based studies show that total psychopathy scores are associated with both reactive and proactive aggression, though the contributing facets vary (Blais et al., 2014; Cima & Raine, 2009). Facet-level analyses have revealed that interpersonal and affective traits more strongly predict proactive aggression, while impulsive and antisocial traits are often linked to reactive aggression, particularly among individuals with poor emotion regulation (Thomson et al., 2018, 2021). These associations underscore the importance of disaggregating psychopathy to understand how specific traits confer risk for different forms of aggressive behavior.

While psychopathy facets show distinct associations with aggression subtypes, their links to more severe forms of violence are less consistent. Studies in offender populations suggest the interpersonal facet often predicts proactive violence (Blais et al., 2014; Declercq et al., 2012), but findings on the antisocial facet are mixed, with some identifying it as a key contributor (Thomson, 2017; Thomson et al., 2016, 2019; Walters & Heilbrun, 2010), and others finding no association (Declercq et al., 2012; Mossière et al., 2020). The affective facet also yields conflicting results, with studies linking it to both increased (Mossière et al., 2020; Thomson et al., 2016) and decreased (Walsh et al., 2009) risk for proactive violence. These inconsistencies may stem from studies failing to distinguish between reactive and proactive forms of violence. For instance, Sohn et al. (2022) found proactive homicides were more closely tied to affective traits, whereas reactive homicides aligned with the antisocial facet. Such findings underscore that psychopathy’s influence on violence varies by context and motivation, not just trait expression.

Recent studies have begun to examine how psychopathy’s facets relate to gun use in violent crime, revealing important findings. Kjærvik and Thomson (2024) found that unpermitted gun carrying and self-defense gun use were associated with higher antisocial facet scores, and both the affective and antisocial facets were positively associated with firearm violence. A study involving offenders found gun violence-related crimes were perpetrated by offenders scoring higher on interpersonal and affective traits when compared to general violent offenders (Thomson, 2022). Between these two studies, there are consistencies with gun violence being related to the affective facet and inconsistencies with gun violence being related to the interpersonal and antisocial facet. This is likely explained by the studies not accounting for the motivation or subtype of gun violence. As found with aggression and severe forms of violence, we would expect the psychopathy facets to be differentially related to gun violence subtypes, which is important for informing gun violence prevention strategies (Thomson et al., 2025).

Most studies examining the relationship between psychopathy, aggression, and violence focus on student samples or incarcerated populations, leaving a critical gap in research on *high-risk community* samples, such as violently injured patients. Studying this population is particularly important because they are at increased risk for future firearm violence but live in the community, providing a unique opportunity to examine the dynamics of gun violence in a non-institutionalized, high-risk population (Cunningham et al., 2015; Hartman et al., 2024; Kevorkian & Thomson, 2024). By focusing on violently injured individuals, this study fills a critical gap in firearm violence research, offering real-world insights into risk factors and intervention opportunities that are directly applicable to healthcare-based prevention efforts.

The aim of the present study was to understand the relation between psychopathy and aggression and gun violence subtypes. We chose to examine both total psychopathy scores and facet-level scores to balance parsimony and precision. Total scores offer a broad overview of psychopathy’s relationship with aggression and gun violence, particularly given the generally high correlations among facets, which may suggest shared variance. However, facet-level analyses allow for a more nuanced understanding of how specific psychopathy facets may differentially contribute to distinct forms of aggression and gun violence. This dual approach provides a comprehensive view of psychopathy’s role in violence while preserving the ability to detect unique facet-specific relationships that are critical for targeted prevention strategies.

To assess the facet-level associations, we first assessed the factor structure of the Self-Report Psychopathy Scale. Using the 4-facet model of psychopathy and total scores of psychopathy, we explored the associations between reactive and proactive aggression, and reactive and proactive gun violence. Drawing on past research, although mixed, we expected psychopathy total scores to be positively related to all forms of aggression and gun violence. For the facet-level associations, we expected the interpersonal facet to be related to proactive aggression, and the antisocial facet to be related to reactive aggression. We expected both the interpersonal and affective facets to be related to proactive gun violence, and the antisocial facet to be related to both reactive and proactive gun violence.

## Method

### Participants

This study included 458 violently injured adults, primarily male (72%), aged 18 to 75 years (*M* = 32.76, *SD* = 12.79). Most participants self-identified as Black/African American (78%), followed by White/Caucasian (16%), Asian (0.7%), American Indian (0.2%), or other (5.1%), with 75.4% identifying as non-Hispanic. Regarding injuries leading to hospitalization, 53% had sustained gunshot wounds, 8% stab wounds, and 39% assault wounds, while 21.6% reported prior hospitalization for violence-related injuries. Regarding education and employment, 79% had completed 12th -grade education or earned a GED, and 42% were employed or on temporary leave due to injury. The present study had a recruitment rate of 76%.

## Procedure

The sample was recruited from an urban Level 1 Trauma Center in Virginia, either through the inpatient setting or the emergency department, as part of a larger project on retaliatory gun violence (R01CE003296, PI: Thomson). Patients were eligible to participate if they were > 18 years old, had sustained a violence-related injury, and were not prisoners. The population focus of the present study is people who were violently injured, as violently injured patients are at high risk for future violence and violence-related re-injury. Before consent, participants were informed that their involvement in the study was for research only and that involvement would not impact their medical care. Participants completed the Self-Report Psychopathy Scale at baseline assessment and were compensated $160 for participating in the larger project. The study was approved by the Virginia Commonwealth University institutional review board and received a Certification of Confidentiality from the Centers for Disease Control and Prevention.

## Measures

### Psychopathy

The Self-Report Psychopathy – Short Form (SRP-SF; Paulhus et al., 2016) consists of 29 items designed to measure the four facets of psychopathy: Interpersonal (7 items, e.g., “I have pretended to be someone else in order to get something”), Affective (7 items, e.g., “I don’t feel bad about hurting people”), Lifestyle (7 items, e.g., “I’ve often done something dangerous just for the thrill of it”), and Antisocial (7 items, e.g., “I have tricked someone into giving me money”). Items were rated on a 5-point Likert scale (1 = disagree strongly to 5 = agree strongly). Scores ranged from 1 to 5, with higher scores indicating increased levels of psychopathy. The total scale and subscales showed good validity in the present study (Total, α = 0.93; Interpersonal, α = 0.83; Affective, α = 0.77; Lifestyle, α = 0.82; Antisocial, α = 0.78).

### Reactive-Proactive Aggression

The Reactive-Proactive Questionnaire (RPQ) is a self-report inventory of aggression divided into two functions of aggression: reactive (11 items, e.g., “Reacted angrily when provoked by others”) and proactive (12 items, e.g., “Hurt others to win a game”). Items were rated on a 3-point Likert scale (0 = never to 2 = often). The subscales showed good validity in the present study (Reactive, α = 0.86; Proactive, α = 0.88).

### Reactive-Proactive Gun Violence

The Gun Violence Questionnaire (GVQ) is a self-report assessment of gun violence with two functions of gun violence: reactive (6 items, e.g., “Shot a gun at others when teased”) and proactive (8 items, e.g., “Used a gun to take things from other people”). Scores were coded as 0 = No or 1 = Yes.

### Firearm Access

Consistent with prior research (Kjærvik & Thomson, 2024; Richards et al., 2021), firearm access was measured using a single item to assess gun access: “Could you get access to a firearm if you wanted?” (response options: yes, no).

## Statistical Analyses

First, descriptive statistics and correlations were examined, as displayed in Table 1. Next, a Confirmatory Factor Analysis (CFA) was conducted in R version 4.1.1 (R Core Team, 2024) using the *lavaan* package (Rosseel, 2012) to evaluate the model fit. Items of the SRP were loaded on the four dimensions as specified previously (Paulhus et al., 2016): affective (items 3, 8, 13, 16, 18, 24, and 28), lifestyle (items 1, 4, 11, 14, 17, 21, and 27), interpersonal (items 7, 9, 10, 15, 19, 23, and 26), and antisocial (items 5, 6, 12, 20, 22, 25, and 29). The assessment of model fit was conducted using an absolute index (Root Mean Square Error of Approximation, RMSEA) and incremental indexes (Comparative Fit Index, CFI; Tucker Lewis Index, TLI; Goodness of Fit, GFI; Standardized Root Mean Square Residual, SRMR). Models were estimated using the Diagonally Weighted Least Squares (DWLS) estimation, which is most appropriate for use with ordinal items (Flora & Curran, 2004). Because not all model fit indices are stable across model specifications, multiple fit indices were reported, and the following criteria were applied (CFI > 0.95, TLI > 0.95, GFI > 0.95, RMSEA < 0.07, and SRMR < 0.08; Kline, 2016). Additionally, several indices were used to assess the reliability and validity of the SRP: SF factor structure beyond standard CFA model fit. Composite Reliability (CR) and Average Variance Extracted (AVE) were used to assess convergent validity, with CR values above 0.70 and AVE values above 0.50 considered acceptable. Discriminant validity was assessed using the Maximum Shared Variance (MSV), with MSV values lower than the corresponding AVE values indicating that each construct captures more variance from its items than it shares with other constructs (Hair et al., 2010). The heterotrait-monotrait ratio of correlations (HTMT) was also used to assess discriminant validity, with values below 0.90 suggesting that the constructs are empirically distinct (Henseler et al., 2015; Roemer et al., 2021). Including these indices provides a more nuanced evaluation of measurement quality than CFA fit indices alone, as they capture multiple aspects of construct validity. Specifically, how well items represent their intended latent factor (convergent validity), how distinct each factor is from others (discriminant validity), and the internal consistency of each construct (reliability).

**Table 1:** Correlations between study variables and descriptive statistics

Measures	1	2	3	4	5	6	7	8	9	10	11	12
1. Sex^a^	-											
2. Age	0.06	-										
3. Gun access^a^	0.12*	0.08	-									
4. Reactive AG	− 0.03	− 0.11*	0.12*	-								
5. Proactive AG	0.06	− 0.03	0.10*	0.63**	-							
6. Reactive GV^a^	0.14*	− 0.05	0.16**	0.38**	0.41**	-						
7. Proactive GV^a^	0.11*	0.03	0.09	0.33**	0.46**	0.65**	-					
8. Interpersonal	0.11*	− 0.03	0.07	0.44**	0.55**	0.33**	0.26**	-				
9. Affective	0.10*	− 0.13*	0.10*	0.58**	0.53**	0.38**	0.30**	0.68**	-			
10. Lifestyle	0.11*	− 0.06	0.11*	0.57**	0.55**	0.36**	0.31**	0.69**	0.72**	-		
11. Antisocial	0.15*	0.008	0.16**	0.49**	0.59**	0.45**	0.44**	0.71**	0.64**	0.69**	-	
12. SRP Total	0.13*	− 0.06	0.12*	0.60**	0.64**	0.43**	0.37**	0.87**	0.87**	0.89*	0.86**	-
*M*				7.32	2.20	0.20	0.11	12.15	15.20	14.22	12.19	53.75
*SD*				4.42	3.58	0.40	0.32	5.17	5.84	5.84	5.19	19.28
*Skew*				0.42	2.95	1.52	2.42	1.05	0.52	0.72	1.03	0.77
*Kurtosis*				−0.20	11.23	0.31	3.89	1.15	−0.18	0.09	0.71	0.43

Sex (0 = Female, 1 = Male). Gun access (0 = No, 1 = Yes). AG = Aggression. GV = Gun Violence

^a^ Dichotomous variable

*p <.05, **p <.001

Lastly, regression analyses were conducted to examine whether total psychopathy and its four facets were associated with reactive and proactive aggression, as well as gun violence, while controlling for age and sex at birth. Linear regression models with robust estimates were used for aggression outcomes to account for kurtosis in the proactive aggression variable, using the ‘lmtest’ package in R (Zeileis & Hothorn, 2002). Logistic regression models were used for binary gun violence outcomes and were conducted using the base ‘stats’ package in R (R Core Team, 2024).

A priori power analysis in G*Power with effect size = 0.15, alpha = 0.05, power = 95, and 6 predictors indicated that a total sample size of 146 was needed. A total of 488 participants were included in the project. However, 29 participants did not fill out the SRP and were excluded before any analysis. This study included 458 participants, indicating sufficient power.

## Results

### Confirmatory Factor Analysis: Four-Facet Structure

Table 2 displays factor loadings and model fit statistics. The four-facet model of psychopathy showed a good fit, *x*^2^ (344) = 953.085, CFI = 0.989, TLI = 0.988, GFI = 0.987, RMSEA = 0.062, SRMR = 0.064. All factor loadings were significant (*p* <.001) and above 0.5, indicating good indicator reliability. The items with the lowest factor loadings were in the affective facet (e.g., item 28 = 0.53; item 13 = 0.56). The latent variable correlations were high (e.g., interpersonal and antisocial = 0.93), suggesting potential issues with discriminant validity.

**Table 2:** Factor loadings and model fit statistics: Four-Correlated factor model

Item	Interpersonal	Affective	Lifestyle	Antisocial
#7 (false identity)	0.80***			
#9 (enjoy scamming people)	0.85***			
#10 (enjoy pushing people)	0.81***			
#15 (take advantage of others)	0.79***			
#19 (pretend to like people)	0.77***			
#23 (flattery)	0.76***			
#26 (people are easily fooled)	0.75***			
#3 (people are weak)		0.66***		
#8 (enjoy watching fights)		0.76***		
#13 (do not keep in touch with family)		0.56***		
#16 (cold-hearted)		0.72***		
#18 (enjoy violent movies and sports)		0.66***		
#24 (do not feel bad about hurting others)		0.76***		
#28 (dump friends when not useful)		0.53***		
#1 (rebellious)			0.65***	
#4 (thrilled by danger)			0.75***	
#11 (like doing wild things)			0.72***	
#14 (do not follow rules)			0.83***	
#17 (like to have sex with strangers)			0.68***	
#21 (do not learn from mistakes)			0.73***	
#27 (mouth off without thinking)			0.66***	
#5 (have gotten money through trickery)				0.80***
#6 (have assaulted an officer or social worker)				0.78***
#12 (have broken in to steal or vandalize)				0.78***
#20 (have been convicted of serious crime)				0.62***
#22 (carry weapon sometimes for protection)				0.61***
#25 (have used threats)				0.85***
#29 (have attacked someone intentionally)				0.77***
*x*^*2*^ *Test of Model Fit*	953.085, *df* = 344, *p* <.001			
CFI				0.989
TLI				0.988
GFI				0.987
RMSEA	0.062, 90% CI [0.058, 0.067]			
SRMR				0.064

***p <.001, **p <.01, *p <.05

Table 3 displays the convergent and discriminant validity estimates. The CR was acceptable for all factors (> 0.70), indicating good internal consistency. However, the AVE for the affective facet was 0.45, which is below the 0.50 threshold, suggesting limited convergent validity. There is a potential issue with discriminant validity due to high latent correlations (e.g., MSV > AVE). For example, the MSV = 0.865 for interpersonal and antisocial, indicating shared variance between constructs. HTMT values were below 0.90, which supports discriminant validity, although some constructs (e.g., interpersonal and antisocial) were close to the threshold.

**Table 3:** Self-Report psychopathy: convergent and discriminant validity

				HTMT			
	CR	AVE	MSV	1	2	3	4
Acceptable fit	> 0.70	> 0.50	< AVE	< 0.90			
1. Interpersonal	0.912	0.624	0.865	-	0.84	0.85	0.87
2. Affective	0.813	0.447	0.847	0.87	-	0.90	0.81
3. Lifestyle	0.855	0.516	0.847	0.87	0.92	-	0.86
4. Antisocial	0.832	0.560	0.865	0.93	0.85	0.88	-

*CR *Composite Reliability, *AVE *Average Variance Extracted, *MSV *Maximum Shared Variance, *HTMT *Heterotrait-heteromethod correlations

Bottom half of correlation matrix is bivariate correlations. Top half of correlation matrix is multivariate correlations

### Psychopathy and Reactive-Proactive Aggression

Table 4 displays the results of the multiple linear regressions. First, total psychopathy scores were significantly associated with reactive aggression, *F*(4, 454) = 103.4, *p* <.001. In this model, psychopathy (*p* <.001), sex (*p* =.001), age (*p* =.03), and proactive aggression (*p* <.001) were significantly related to reactive aggression. When examining psychopathy facet scores, the model remained significant, *F*(7, 451) = 68.5, *p* <.001. The interpersonal (*p* =.003), affective (*p* <.001), and lifestyle facets (*p* <.001), along with sex (*p* =.005), were significantly related to reactive aggression. The interpersonal facet showed a small effect size, as indicated by the confidence interval’s proximity to zero, [−0.23, −0.04], and its negative direction may indicate a potential suppression effect (see Table 4). Suppression effects can arise when multiple predictors in a statistical model are highly correlated. Post-hoc analysis confirmed that the interpersonal facet was nonsignificant when entered without the other facets, indicating a suppression effect.

**Table 4:** Linear regression with robust estimates: psychopathy predicting reactive and proactive aggression (AG)

	Reactive Aggression						Proactive Aggression					
	*B*	*SE B*	$\:\beta\:$	95% CI		∆*R*^*2*^	*B*	*SE B*	$\:\beta\:$	95% CI		∆*R*^*2*^
				LL	UL					LL	UL	
**Model**						0.47***						0.50***
Sex	−0.96	0.33	−0.10**	−1.61	−0.32		0.20	0.29	0.03	−0.37	0.77	
Age	−0.03	0.01	−0.08*	−0.05	−0.005		0.01	0.01	0.04	−0.006	0.03	
AG	0.52	0.06	0.42***	0.40	0.64		0.32	0.04	0.40***	0.24	0.41	
SRP Total	0.08	0.01	0.34***	0.05	0.10		0.07	0.01	0.40***	0.05	0.09	
**Model**						0.51***						0.51***
Sex	−0.93	0.32	−0.09**	−1.56	−0.30		0.19	0.28	0.02	−0.37	0.74	
Age	−0.02	0.01	−0.05	−0.04	0.002		0.007	0.01	0.03	−0.01	0.02	
AG	0.53	0.06	0.43***	0.43	0.64		0.35	0.05	0.43***	0.25	0.44	
Interpersonal	−0.14	0.05	−0.16**	−0.23	−0.04		0.13	0.05	0.19***	0.03	0.23	
Affective	0.22	0.04	0.29***	0.13	0.30		−0.01	0.03	−0.02	−0.08	0.05	
Lifestyle	0.17	0.04	0.22***	0.08	0.25		0.02	0.04	0.03	−0.06	0.10	
Antisocial	0.02	0.05	0.03	−0.07	0.12		0.16	0.05	0.24***	0.07	0.26	

Sex (0 = Female, 1 = Male). AG = either reactive or proactive aggression depending on the model. *CI *Confidence Interval, *LL *Lower Limit, *UL *Upper Limit

***p <.001, **p <.01, *p <.05, ^†^*p* <.10

In the proactive aggression model, total psychopathy was significantly related to proactive aggression, *F*(4, 454) = 115.1, *p* <.001. Both psychopathy (*p* <.001) and reactive aggression (*p* <.001) were significantly related to proactive aggression. The facet-level model was also significant, *F*(7, 451) = 70.19, *p* <.001, and the interpersonal (*p* <.001) and antisocial facets (*p* <.001), along with reactive aggression (*p* <.001), were significantly associated with proactive aggression.

### Psychopathy and Reactive-Proactive Gun Violence

Table 5 displays the results of the logistic regression models. Total psychopathy was significantly related to reactive gun violence, χ²(5) = 198.52, *p* <.001, indicating that the predictors distinguished between individuals with and without reactive gun violence. This model explained 56.1% of the variance (Nagelkerke R² = 0.561) and 35.5% based on Cox & Snell R². McFadden’s R² (0.44) suggested a strong fit relative to the null model. The likelihood ratio test confirmed a significant improvement in model fit over the null model (ΔLogLik = −99.26, *p* <.001). Total psychopathy (*p* <.001), proactive gun violence (*p* <.001), and gun access (*p* =.01) were all positively related to reactive gun violence.

**Table 5:** Logistic regression: psychopathy predicting reactive and proactive gun violence (GV)

	Reactive Gun Violence					Proactive Gun Violence				
	*B*	*SE B*	OR	95% CI		*B*	*SE B*	OR	95% CI	
				LL	UL				LL	UL
**Model**										
Sex	0.47	0.42	1.60	0.72	3.84	0.66	0.59	1.94	6.37	6.52
Age	−0.03	0.02	0.97^†^	0.94	1.00	0.04	0.02	1.04^†^	10.00	1.08
Gun access	0.86	0.34	2.36*	1.22	4.70	−0.13	0.43	8.77	3.78	2.02
GV	4.21	0.58	67.05***	23.90	244.32	4.30	0.60	7.37***	2.56	2.78
SRP Total	0.04	0.009	1.04***	1.03	1.06	0.03	0.01	1.03**	1.01	1.06
**Model**										
Sex	0.47	0.42	1.59	0.72	3.85	0.34	0.61	1.40	0.44	4.99
Age	−0.03	0.02	0.97	0.94	1.00	0.04	0.02	1.04	1.00	1.08
Gun access	0.87	0.34	2.39*	1.23	4.79	−0.39	0.46	0.67	0.27	1.65
GV	4.20	0.60	66.60***	22.92	250.19	4.28	0.61	71.91***	24.36	279.15
Interpersonal	0.03	0.05	1.03	0.94	1.12	−0.07	0.06	0.94	0.83	1.04
Affective	0.10	0.04	1.10*	1.01	1.20	0.02	0.06	1.02	0.91	1.14
Lifestyle	0.003	0.04	1.00	0.92	1.09	−0.02	0.06	0.99	0.88	1.10
Antisocial	0.06	0.05	1.06	0.97	1.16	0.23	0.06	1.26***	1.12	1.44

Sex (0 = Female, 1 = Male). Gun Access (0 = No, 1 = Yes). GVQ = either reactive or proactive gun violence depending on the model. OR = Odds ratio. CI = Confidence Interval, LL = Lower Limit, UL = Upper Limit

***p <.001, **p <.01, *p <.05, ^†^*p* <.10

The model including psychopathy facets was also significant, χ²(8) = 200.35, *p* <.001, demonstrating that these predictors collectively distinguished between those with and without reactive gun violence. This model accounted for 56.54% of the variance (Nagelkerke R² = 0.565) and 35.81% based on Cox & Snell R², with McFadden’s R² (0.442) indicating a good fit compared to the null model. The likelihood ratio test supported a significant improvement over the null model (ΔLogLik = −100.17, *p* <.001). Among the predictors, the affective facet (*p* =.02), gun access (*p* =.01), and proactive gun violence (*p* <.001) significantly predicted reactive gun violence, indicating that higher affective characteristics, gun access, and proactive gun violence, were associated with increased odds of reactive gun violence.

For proactive gun violence, the total psychopathy model was also significant, χ²(5) = 171.66, *p* <.001, explaining 61.8% of the variance (Nagelkerke R² = 0.618) and 31.6% based on Cox & Snell R². McFadden’s R² (0.53) suggested a strong fit relative to the null model. The likelihood ratio test confirmed a significant improvement in model fit over the null model (ΔLogLik = −85.83, *p* <.001). Total psychopathy (*p* =.005) and reactive gun violence (*p* <.001) were positively related to proactive gun.

The facet-level model was also significant, χ²(8) = 184.29, *p* <.001, demonstrating that the predictors collectively distinguished between those with and without proactive gun violence. This model explained 65.45% of the variance (Nagelkerke R² =0.655) and 33.48% based on Cox & Snell R². According to McFadden’s R² (0.569), the model demonstrated a strong fit compared to the null model. The likelihood ratio test confirmed that the full model significantly improved fit over the null model (ΔLogLik = −92.15, *p* <.001). The antisocial facet (*p* <.001) and reactive gun violence (*p* <.001) were positively related to proactive gun violence, suggesting that higher antisocial characteristics and reactive gun violence were associated with increased odds of proactive gun violence.

## Discussion

The present study examined the association between psychopathy total scores and its facets with subtypes of aggression and firearm violence among a high-risk violently injured community sample. In line with our hypotheses, psychopathy was associated with both proactive and reactive forms of aggression and gun violence. We also partially confirmed our hypotheses for the facets of psychopathy, with the affective and lifestyle facets being associated with reactive aggression, while the interpersonal and antisocial facets predicted proactive aggression. We expected both the interpersonal and affective facets to be related to proactive gun violence, and the antisocial facet to be related to both reactive and proactive gun violence. However, we found that only higher affective facet scores were related to reactive gun violence, and higher antisocial facet scores were related to proactive gun violence. These findings, while partially consistent with prior theory, reveal distinct associations between psychopathy facets and specific violence outcomes. Given the relatively limited research on psychopathy and firearm violence in community samples, especially among high-risk populations, these results offer new insights that may help inform more targeted intervention and prevention strategies.

In line with our expectations for proactive aggression, the interpersonal facet, which reflects superficial charm, manipulativeness, and deceitfulness, was associated with proactive aggression, a finding consistent with Blais et al.’s (2014) meta-analysis involving 53 studies. Proactive aggression is a strategic and goal-oriented behavior. Individuals high in interpersonal psychopathic traits are adept at using charm and deception to control others, allowing them to engage in premeditated aggression to achieve power, financial gain, or dominance (Raine et al., 2006). We also found that the antisocial facet was related to proactive aggression, which supports prior research in community samples (Thomson et al., 2018) but is different from findings among violent offenders (see Declercq et al., 2012). Nevertheless, these results are not surprising as having higher antisocial facet scores is indicative of greater criminal versatility, criminal thinking styles, and a more extensive history of antisocial behavior (Olver & Wong, 2015). This finding also aligns with longitudinal research showing that antisocial traits from adolescence into young adulthood are associated with chronic offending, legal system involvement, and long-term impairment (Salekin, 2008), further underscoring the relevance of antisociality in predicting goal-directed violence.

Our results for reactive aggression were somewhat different than expected, with both lifestyle and the affective facet emerging as the only predictors. The lifestyle facet, which encompasses impulsivity, irresponsibility, and sensation-seeking, aligns with theories suggesting that reactive aggression is driven by poor self-regulation and an inability to inhibit emotional responses to perceived threats or provocations (Raine et al., 2006; Thomson, 2019). This finding is also consistent with prior research indicating that individuals high in lifestyle psychopathic traits are more likely to engage in impulsive and reactive forms of aggression (Blais et al., 2014; Thomson et al., 2018). The affective facet, on the other hand, which includes traits such as callousness, a lack of empathy, shallow affect, and an absence of remorse or guilt, is often conceptually linked to proactive violence (Sohn et al., 2022) and considered to support “cold-blooded” perpetration (DeLisi, 2016), which we did not find. Our finding linking affective facets to reactive aggression may be explained by poor emotion regulation, a pattern observed in research on psychopathy and aggression (Garofalo et al., 2020; Thomson et al., 2018). That is, individuals with high affective psychopathy traits may struggle to regulate emotional responses, leading to inappropriate, exaggerated aggression when provoked. Additionally, deficits in social cognition, such as poor emotion recognition, may contribute to affective psychopathic traits in relation to reactive aggression (Igoumenou et al., 2017; Remmel et al., 2024). Recent research has found that youth high on affective traits not only have deficits in emotion recognition but also have a tendency to interpret fearful faces as angry (Ciucci et al., 2024). Therefore, affective traits associated with reactive aggression and gun violence may be due to social information processing deficits. However, this study did not test mediators/moderators explaining this association, but the present findings are an important first step in understanding potential gun violence prevention strategies.

The interpersonal facet was also significant in the reactive aggression model but showed a small negative association. Follow-up analyses indicated this was a suppressor effect. Although the interpersonal facet was positively correlated with reactive aggression at the bivariate level, it became negatively associated only when the other psychopathy facets were included in the model. This pattern suggests that the interpersonal facet shares variance with more predictive traits, such as the affective and antisocial facets, and that its unique variance may not meaningfully relate to reactive aggression in this sample. Rather than indicating a protective effect, the negative coefficient likely reflects a statistical artifact. This highlights the interpretive challenges that emerge when predictors are highly intercorrelated, particularly in facet-level models where overlapping constructs compete to explain shared variance.

Gun violence is among the most severe forms of violence due to its high lethality and long-term societal impact (Aboutanos et al., 2025; Thomson, 2022), making it a critical focus for violence prevention and public health efforts (Hartman et al., 2024; Sullivan et al., 2024). Indeed, gun violence is one of the leading causes of death (Fowler et al., 2025) and injury in the U.S (Holland et al., 2025), making it one of the costliest public health issues (Roche et al., 2025). Our findings highlight distinct relationships between psychopathy facets and firearm violence, revealing notable parallels to the aggression analyses. Consistent with the results for reactive aggression, reactive gun violence was uniquely associated with higher affective facet scores but not lifestyle facet scores. Similarly, in line with proactive aggression, proactive gun violence was linked to higher antisocial facet scores but no significant association with the interpersonal facet. These findings suggest that both the affective and antisocial facets of psychopathy contribute to aggression versatility, even in the most severe forms of violence involving firearms. In contrast, while the lifestyle and interpersonal facets may indicate a general risk for aggression, their influence appears less pronounced in firearm-related violence, suggesting that their role may be more limited to non-lethal forms of violence. This is an important distinction for informing gun violence prevention frameworks.

Identifying different risk factors for types of firearm-related violence provides specific targets for prevention strategies across contexts. Effective gun violence prevention programs must go beyond broad approaches and be tailored to the individual’s needs, especially when engaging high-risk individuals (Thomson, 2024). This includes approaches that target psychological traits that are well-known to drive different forms of firearm violence (Mehari et al., 2025). By demonstrating that only the affective and antisocial facets of psychopathy are related to firearm violence, this study may help inform future efforts aiming to enhance risk assessment and prevention programs for gun violence. For example, when preventing retaliatory gun violence, targeting the drivers of affective psychopathic traits may be beneficial, especially as a prevention strategy among youth (Kevorkian & Thomson, 2024). In contrast, goal-directed gun violence may be prevented through strategies targeting criminal thinking styles.

Existing hospital-based violence intervention programs (HVIPs), which provide support to violently injured patients, may need to incorporate more targeted approaches focused on reducing retaliation motives among individuals with high affective and potential deficits associated with affective psychopathic traits (e.g., emotion recognition, emotion regulation, hostile attributional biases). Psychoeducational programs addressing moral disengagement, perspective-taking, and alternative conflict resolution strategies could help disrupt the cycle of retaliatory shootings in this population. In contrast, proactive gun violence prevention efforts may benefit from focusing on antisocial facet characteristics (e.g., use of violence for gain, criminal versatility, community-based deterrence and intervention programs, including focused crisis intervention strategies from case managers with lived experiences from communities [e.g., Violence Interrupters]). Collectively, this may be particularly effective for individuals with high antisocial traits, as it combines targeted law enforcement with case managers who have lived experiences with structured social and economic support.

### Limitations

While this study offers important insights, it also has several limitations. First, the cross-sectional design prevents causal inferences about the relationship between psychopathy facets, aggression, and gun violence. Longitudinal research is needed to examine how psychopathy predicts future firearm violence and aggression over time. Second, the generalizability of findings is limited by the nature of the sample, which is composed of violently injured, high-risk individuals recruited from a single urban hospital. While this population is highly relevant for understanding firearm violence in real-world settings (Thomson & Kevorkian, 2024), the findings may not extend to incarcerated populations, who are removed from community violence contexts, or to lower-risk groups such as university students, among whom firearm violence is less prevalent. Notably, this sample is at increased risk for retaliatory violence (Thomson et al., 2025), which offers a unique lens into risk factors operating in real time at a critical moment, often described as a “window of opportunity” for interrupting the cycle of community violence (Aboutanos et al., 2025). Third, although all participants were informed that the study was protected by a Certificate of Confidentiality, we cannot rule out the possibility that fear of retaliation, legal consequences, or social stigma may have influenced the accuracy of their self-reports, particularly regarding firearm-related behaviors. Given their recent victimization, some participants may have felt ashamed, suspicious, or even vengeful, all of which could shape disclosure. These concerns are especially relevant in interpreting sensitive items related to gun use and aggression. Fourth, measurement-related limitations should be acknowledged. At the facet level, multicollinearity among psychopathy dimensions introduces the risk of shared variance distorting the interpretation of individual effects. Suppression effects, particularly with the interpersonal facet, further complicate these models.

Another limitation concerns the item content within the SRP-SF facets. Although our confirmatory factor analysis supported the four-factor model and demonstrated acceptable reliability and validity, some items may reflect content that overlaps thematically with other psychopathy dimensions. For example, the antisocial item “tricked someone into giving me money” could also reflect interpersonal manipulation, and certain items referencing violence or weapon carrying (e.g., “I have assaulted a law enforcement official or social worker”; “Every now and then I carry a weapon for protection”) may resemble behavioral outcomes assessed in this study. While these items capture relevant aspects of psychopathy, they may contribute to associations with non-specific subtypes of aggression or firearm violence outcomes through shared content rather than distinct psychological processes.

## Conclusion

In conclusion, this study highlights the importance of psychopathy as a multidimensional construct in predicting aggression and firearm violence subtypes. The findings emphasize the need to consider psychopathy’s distinct facets when developing risk assessments and intervention strategies for aggression and gun violence. Additionally, recognizing psychopathy’s relevance beyond forensic settings, particularly in hospital and healthcare injury prevention contexts, may further advance violence prevention efforts in high-risk populations.

## Declarations

This study was not pre-registered. The data presented in this study may be made available upon request from the corresponding author. The data are not publicly available due to the potential for personal identification of participants in the present sensitive population.
